# Changing the long-term care spectrum

**DOI:** 10.1186/s12877-022-02909-8

**Published:** 2022-04-08

**Authors:** Hilde Verbeek, Gary Mitchell

**Affiliations:** 1 Department of Health Services Research, Care and Public Health Research Institute, Maastricht University, Maastricht, The Netherlands; 2 School of Nursing & Midwifery, Queen’s University Belfast, Belfast, Northern Ireland UK

Our population is ageing rapidly, impacting the way we live, dwell, care, and work with each other in society. Older persons have a higher chance of developing diseases, especially related to neurodegenerative diseases such as dementia and do not only influence physical functioning but also lead to disabilities in mental and social health. Concerningly, the group older people who require intensive and continuous care is growing significantly, whilst at the same time, the group of people who are available to care for them is decreasing [5]. With labor markets for health and social care experiencing tremendous shortages and the demand for well-educated and trained staff increasing, the group of people providing the vast majority of informal care (aged 45–60) is decreasing rapidly. Furthermore, with trends of retreating governments expenditure on costs of care and service provision increasingly being paid by older people themselves [35] the demand for high-quality long-term care services sees a continued rise.

These developments highlight the need to redesign long-term care for older people dramatically. Long-term care services support people to maintain or improve their functioning in daily life and quality of life [19]. It can include both health and social care related support services and takes place in various settings (e.g. home, within the community or in facilities). Supported by a cultural change movement, a fundamental shift in thinking about long-term care environments has emerged [34]. Increasingly older people wish to remain in their own home and age-in-place [20]), with research showing living at home to be an essential part of an individual’s identity and social network, relating to positive feelings, memories and well-being [4]. However, as a barrier to aging in place a substantial proportion of older people will become frail, develop chronic and complex diseases and experience intensive long-term needs for care, such as day-care or 24 h care in nursing homes. With the expectation of a continued increase in the size of this population, long-term care facilities (e.g. care homes, nursing homes, assisted living facilities) progressively aim to provide care and service delivery in a home-like environment, thus, supporting normal daily life [28]. Values such as autonomy, retaining one’s own identity and meaningful engagement in activities and social networks are key. Technology, including e-health applications, home electronics and robotics play an important role in enabling these goals, supporting residents’ autonomy, and facilitating staff [31].

Current evidence indicates that traditional long-term care facilities are often not effective in supporting everyday functioning and may even be harmful [15, 28]. Traditional care environments are often associated with poor outcomes for residents, including inactivity, high levels of neuropsychiatric symptoms (e.g. agitation and depression),use of physical restraints and high levels of psychotropic drugs [6, 7, 12, 17, 21, 22]. As a result, alternative care environments are urgently needed, using enabling environmental design to promote health and well-being of older people with complex conditions. Especially for people with dementia, the care environment plays a crucial role to support their daily functioning and well-being, for example when the disease progresses and 24 h care is required. Behaviour and everyday functioning are the result of an interaction between the individual and her/his environment (Lawton et al. 1970 [23]; Lewin 1951 [24]). Depending on the scientific discipline scholars take, long-term care environments have been described (Charras et al. 2016 [8]) as 1) being therapeutic or rehabilitative, focusing on compensating for existing deficits (mainly geriatrics and health sciences origin); (Chaudhury et al. 2014 [9]; Zeisel et al. 1994 [36]) 2) needs-based, focusing on how the environment can meet the needs of people with dementia (rooted in nursing science and psychology (Algas et al. 1996 [2]; Morgan et al. 1999 [26]) or 3) or experience-based, focusing on how people pose meaning to a place through interaction with their environment (i.e. gerontology and social ecology) (Davis et al. 2009 [10], Molony et al. 2011 [27]). Following these descriptions, researchers have aimed to develop evidence-based interventions to improve the well-being and daily life of residents with dementia, especially in traditional nursing homes, with mixed results (Charras et al. 2016 [8]). What often is neglected in practice and research is that congruence is needed between the different environmental components (physical, social and organisational), in order to promote well-being and adapted behaviours for people with dementia and their caregivers (de Boer 2021 [11]). Current long-term care environments may also have highly task-oriented organisational approaches. A mechanism is needed for long-term care environments to adapt, adjust or reconfigure their resource base in response to changing environmental conditions, so-called dynamic capabilities of an organisation (Pablo et al. 2007 [29]). Evidence suggests that shared values and supportive leadership for staff help in setting priorities and improve the delivery of person-centred care (Backman et al. 2020 [3]; Edvardsson 2011 [16]; van Beek et al. 2010 [32]).

More knowledge is highly warranted to disentangle environmental working mechanisms that influence daily functioning in older people in their real-life context, especially for those with dementia. This will increase the effectiveness of quality improvement initiatives and interventions. Furthermore, it will inform practitioners and policy makers in the evidence-based design of care environments for older people. An interdisciplinary approach is needed, synthesizing insights of how the environment is *experienced* (mainly rooted in gerontology and social ecology), how the environment is *used* (mainly rooted in geriatrics, nursing, and psychology), how the environment is *designed* (architecture) and finally how *care services* are provided in the environment (service science and strategic management). Existing theories of functioning mainly focus on biological and/or (psycho) social aspects of ageing and insufficiently consider the interplay of physical, social, and organisational environmental mechanisms. Furthermore, current studies mainly take a deficit-based approach, which ultimately may have negative consequences for functioning in residents’ daily life. It seems more promising to look beyond the disability of older people and focus on their remaining capacities, determining how gains and positive outcomes can be enabled and preserved [14, 25]. This requires that an organisation can continuously adapt to change, and therefore possesses dynamic capabilities in their strategic approach.

Innovative, alternative care environments are being developed that have radically changed long-term care environments. For example, community-based living concepts, green care farms, shared housing arrangements or dementia care villages (De bruin et al. 2017 [13]; Peoples et al. 2020 [30]; Verbeek et al. 2021 [33]). These projects have in common that they aim to provide a homelike environment for older people, encouraging remaining capacity and increasing engagement, autonomy and participation in normal daily life. The role of staff is also changing, with more focus on encouraging remaining capacities, instead of taking over tasks, forming partnerships with family caregivers (Adams et al. 2018 [1]; Gilster et al. 2018 [18]). Furthermore, most projects explicitly aim for embedding within the local community and social networks.

We anticipate that older people requiring long-term care reside in a variety of living arrangements and care concepts in the future. This requires a changing role for all actors involved in the ecosystem of long-term care, including older people, their family care givers, professional caregivers, long-term care providers, social care providers, municipalities, funding agencies, housing organizations and local and national governments.

Long-term care environments can include the home, the community, assisted living facilities, care homes or any facility where people receive long-term care. As such, optimizing the long-term care environment requires the input of multiple disciplines to solve the complex problems highlighted in this article. In this series, we will present original empirical research, evidence synthesis, quality improvement studies and discursive commentaries that move society’s understanding of long-term environments forward. Contributions that consider the relationship between the physical, social, and organizational aspects on the long-term care environment are particularly encouraged. The aim of this collection is to provide high-quality science that can be freely accessed by people who receive or provide care, deliver healthcare education or undertake research in long-term care environments. We hope that this collection will facilitate and empower these individuals to find new ways to optimize care provided within their own long-term care environment.
